# Retinoic acid and meiosis induction in adult versus embryonic gonads of medaka

**DOI:** 10.1038/srep34281

**Published:** 2016-09-28

**Authors:** Mateus C. Adolfi, Amaury Herpin, Martina Regensburger, Jacopo Sacquegno, Joshua S. Waxman, Manfred Schartl

**Affiliations:** 1 University of Wuerzburg, Physiological Chemistry, Biocenter, Am Hubland, D-97074 Wuerzburg, Germany; 2 INRA, UR1037, Fish Physiology and Genomics, Rennes F-35000, France; 3 The Heart Institute, Molecular Cardiovascular Biology and Developmental Biology Divisions, Cincinnati Children’s Hospital Medical Center, Cincinnati, Ohio, USA; 4 Comprehensive Cancer Center Mainfranken, University Clinic Würzburg, Josef Schneider Straße 6, 97074 Wuerzburg, Germany and Texas Institute for Advanced Study and Department of Biology, Texas A&M University, College Station, Texas 77843, USA

## Abstract

In vertebrates, one of the first recognizable sex differences in embryos is the onset of meiosis, known to be regulated by retinoic acid (RA) in mammals. We investigated in medaka a possible meiotic function of RA during the embryonic sex determination (SD) period and in mature gonads. We found RA mediated transcriptional activation in germ cells of both sexes much earlier than the SD stage, however, no such activity during the critical stages of SD. In adults, expression of the RA metabolizing enzymes indicates sexually dimorphic RA levels. In testis, RA acts directly in Sertoli, Leydig and pre-meiotic germ cells. In ovaries, RA transcriptional activity is highest in meiotic oocytes. Our results show that RA plays an important role in meiosis induction and gametogenesis in adult medaka but contrary to common expectations, not for initiating the first meiosis in female germ cells at the SD stage.

In vertebrates, the decision as to whether the bipotential gonad anlage will become testis or ovary is a critical stage in embryonic development. This complex sex determination process includes fate determination and cell differentiation within two fundamentally different programs, the female and the male. These intricate processes are regulated and fine-tuned by cascades or networks of genes. In the past 20 years, the paradigm that ruled the sex determination field was that the genetic machinery controlling gonad development is broadly conserved. Results indicated that the downstream components tend to converge upon the regulation of common effectors, while the ‘master sex-determining genes’, at the top of the genetic hierarchies, show an impressive diversity in different organisms^1^. However, more recent data comparing fish and mammals also indicated discrepancies in the gene expression patterns and in the interactions of the downstream gonadal regulatory network, which mighty reflect a plasticity of this regulatory network to various degrees during vertebrate evolution^2^. In this respect, the development of the gonad is different from all other major vertebrate organs, where generally a high conservation of molecular mechanisms from fish up to humans has been noted^3^. However, despite the modest degree of conservation on the molecular level, there are some commonalities in sexual development of vertebrates.

The undifferentiated gonad of all vertebrates is composed of somatic cells and germ cells, the latter giving rise to the gametes. All gametes originate from primordial germ cells (PGCs), which migrate into the developing gonad^4^. Germ cells are confronted there to take two major decisions; one is the sexual identity of the cell to differentiate as sperm or egg. The other decision is to remain in mitotic division cycles, or to enter into meiosis. The timing of the mitosis/meiosis decision and features of meiosis itself are often different between males and females, suggesting a close relationship between the mitosis/meiosis and sperm/egg decisions^5^. A common feature found in most vertebrates from fish to mammals is an early morphological difference between males and females that becomes apparent on the germ cell level. For instance in medaka, in both XY and XX embryos, the number of PGCs is the same until late embryogenesis. Then PGCs start to proliferate in female embryos and enter meiosis around the hatching stage. In males, it is only at around 15 to 20 days post hatching (dph) the PGCs proliferate again and become spermatogonia^6^. This is a similar situation as in mammals where PGCs in females resume mitosis and enter prophase of the first meiotic division much earlier than in males^7^.

In mammals, it is well known that the critical molecule in the control of meiosis entry is retinoic acid (RA)^8^,^9^. RA is a polar derivative of vitamin A that easily diffuses through tissues and is a classical diffusing morphogen^10^. The balance of the RA-metabolizing enzymes also determines the spatio-temporal distribution of RA^11^. Two of these enzymes are the RA synthesizing enzyme Aldh1a, a member of the retinaldehyde dehydrogenase family (also known as Raldh and can be present in one to several isozymes depending on the species), and the RA-degrading enzymes from the Cyp26/P450-cytochrome family^12^. RA acts as a ligand for nuclear retinoic acid receptors (RARs), which through binding to RA response elements (RAREs) control the expression of RA-responsive genes^13^.

Initial sex differentiation of a mammalian germ cell is not determined by its intrinsic chromosomal constitution, but rather by its cellular environment^14^. This also includes the first entry into meiosis. Expression studies during mouse gonadogenesis indicated that CYP26B1 acts as a meiosis inhibitor degrading RA coming from the mesonephroi to which the gonads are attached^8^,^15^. RA acts to initiate meiosis by inducing expression of the meiosis marker *Stra8* (stimulated by retinoic acid gene 8), followed by the expression of the early meiotic markers *Sycp3* (synaptonemical complex protein 3) and *Dmc1* (dosage suppressor of mck1 homolog)^8^,^16^,^17^.

Although a role of RA in triggering meiosis was mainly described in mammals, evidence shows that this mechanism is likely to be conserved in amphibians and birds^18^,^19^. However, information about such a function in fish is lacking. In zebrafish, recent work based on expression profiles of the RA metabolism enzymes during gonad development and in adults indicated a possible role of RA for meiosis, but no functional information is available so far to confirm this function^20^. Disruption of *ald1a2* in Tilapia leads to a delay in meiosis entry^21^. Analyses of the evolution of *aldh1a* gene family members revealed that teleosts lost some paralogs, and in medaka only the *aldh1a2* gene is still presented^22^. This information together with the fact that no sequence of *stra8* was found thus far in most teleost genomes including medaka, poses the question whether RA has indeed a similar role in meiosis and gonadogenesis in fish as in mammals^23^.

To get a better understanding of the role of RA in meiosis induction in fish we used the model species medaka, were the primary processes of sex determination are reasonably understood. We generated transgenic lines to analyze the transcriptional regulation activity of RA in the gonad during sexual development. Further, we used these tools to analyze the role of RA in the adult gonad of the fish. Our results show that the expression pattern of the RA biosynthesis gene is similar to zebrafish and Tilapia. Studying the timing of production of RA and its activity in the germ cells during development and in the adult gonad of medaka, we show that RA has an important role in meiosis at later stages of gonad development rather than in initial sex determination in the embryo.

## Results

### Synthesis of RA and RA activity during embryonic gonad development

**Figure 1:**
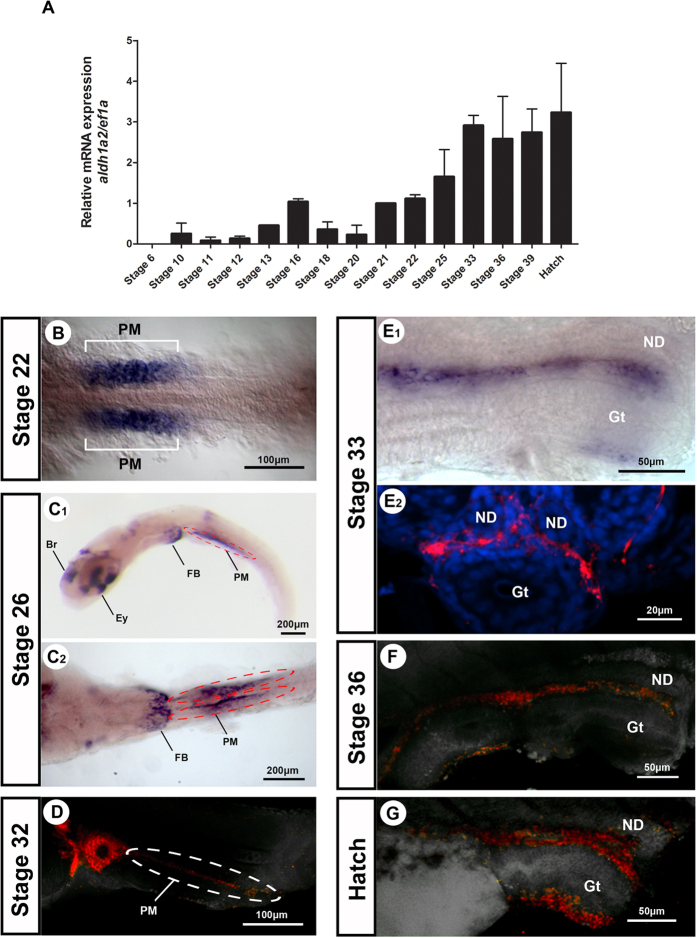
Expression pattern of *aldh1a2* during medaka development. *aldh1a2* (**A**) mRNA expression in embryos of medaka in different stages of development. Values are expressed as arbitrary units of mRNA normalized against the expression levels of *ef1a* amplified from the same template, relative to the expression observed in the stage 21. *In situ* hybridization of *aldh1a2* (**B–G**) in different stages of development. Br, brain; Ey, eye; FB, fin bud; Gt, gut; ND, nephric duct; PM, paraxial mesoderm.

Transcripts levels of *aldh1a2* of different embryonic stages were determined from pooled embryo extracts. Levels are undetectable during early stages but become upregulated around early blastula stage (stage 10) and steadily increase. After stage 33, the levels remain relatively constant until hatching (stage 39) (Fig. 1A).

To identify the source of RA in the gonadal region of the embryo we analyzed expression of *aldh1a2* by *in situ* hybridization (Fig. 1B–G). During early somitogenesis, the gene is strongly expressed in the first four pairs of somites (Fig. 1B). Later, at stage 26, the RNA is still present in the paraxial mesoderm and additionally strongly expressed in the brain, eyes and fin bud (Fig. 1C). From stage 32 until hatching, the expression of *aldh1a2* was detected in cells surrounding the gonad and in the intestine (Fig. 1D–G).

In order to find out if the regions that metabolize RA are also responding to RA, we analyzed responsiveness to RA during embryonic development by means of a transgenic reporter line where 12 copies of the Retinoic Acid Responsive Element (12X RARE) drive eGFP expression. Ubiquitous GFP signals were already observed at early stages of the embryos and could be observed throughout all stages of development (Supplementary Figure 1). When the production of RA was compared to the responsiveness to his molecule, we did not observe any correlation between site of synthesis and transcription regulating activity of RA. For instance, in the eye at stage 26, the expression pattern of *aldh1a2* in the retina is clearly restricted to a ventral and a dorsal domain (Supplementary Figure 2A). In the 12XRARE reporter fish, both domains are present; however, the dorsal part of the retina shows only barely detectable activity (Supplementary Figure 2B). At stage 22, the first four somites present expression of *aldh1a2* (Supplementary Figure 2C); however the neural tube, where *aldh1a2* is not expressed, also showed responsiveness to RA (Supplementary Figure 2D). These results indicate that the RA responsive cells are not restricted to the region where RA is produced and that production of RA is not necessarily connected to transcriptional control activity, in agreement with a non-cell autonomous action of this diffusible signal molecule.

**Figure 2:**
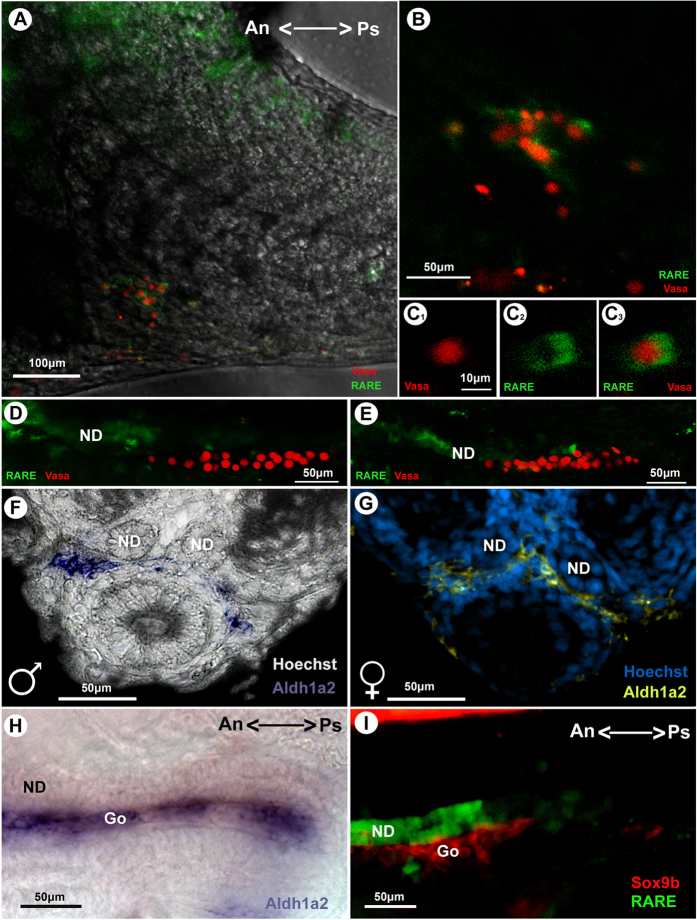
Responsiveness to RA in the gonad during medaka development and correlation with *aldh1a2* expression at stage 33. Responsiveness to retinoic acid (Green) in embryonic stages 30 (**A**–**C**) 35 (**D**) and 37 (**E**) of double transgenic embryos (12XRARE-ef1::gfp::Vasa::H2B::mCherry). Germ cells, vasa positive (Red), at the gonad level. Responsiveness in the germ cells is apparent at stage 30 and switches to the nephric duct at later stages. *In situ* hybridization for *alh1a2* transcripts show expression in the gonadal region in male (**F**) and female (**G**) in transversal section, and lateral view (**H**). Lateral view of double transgenic embryos (12XRARE-ef1::gfp (Green):: sox9b::mCherry (Red)) (**I**) showing no co-localization at this stage. An, anterior region of the embryo; Go, gonadal region; ND, nephric duct; Ps, posterior region of the embryo.

At the gonad level we were able to see GFP positive cells. At the 35 somite stage (stage 30), we identified responsiveness for RA in germ cells of both sexes by crossing the 12XRARE reporter line with Vasa H2B RFP transgenic fish (Fig. 2A–C). Thereafter, no signal was observed in germ cells or in somatic cells until one day after hatching, when the germ cells in females already have entered into meiosis. We observed clear expression of *aldh1a2* in the region of the gonads in both male and female embryos at stage 33 (Fig. 2F,G). Despite expression of *aldh1a2* in the gonads of males and females, the GFP signal indicating RA responsiveness was clearly restricted to the nephric ducts (Fig. 2D,E,H,I). In particular, during the sex determination stages (stage 26 to 36) when meiosis starts in female germ cells^24^, no responsiveness was seen.

### Tissue specific expression of RA metabolism genes in adult medaka

**Figure 3:**
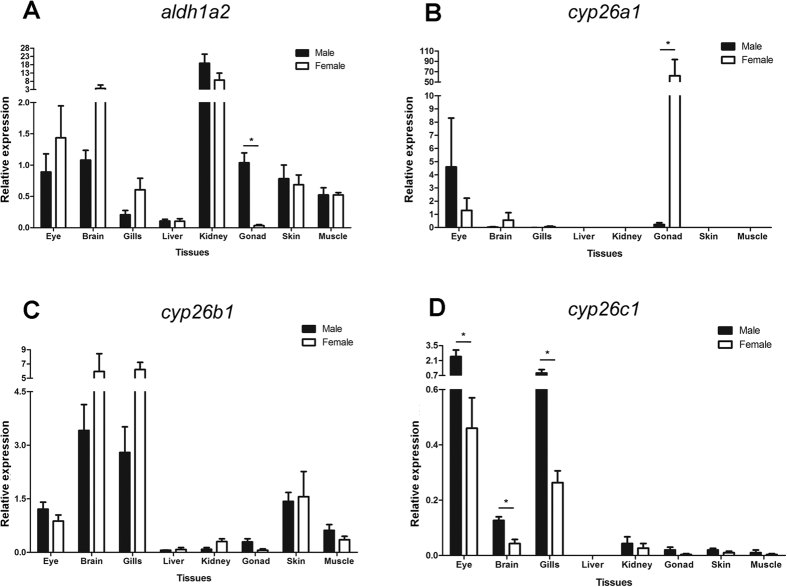
Expression of retinoic acid biosynthesis and degradation enzyme genes in adult medaka. Expression levels of *aldh1a2* (**A**), *cyp26a1*, (**B**), *cyp26b1* (**C**), *cyp26c1* (**D**) in organs of adult male and female medaka determined by qRT-PCR. Values are expressed as arbitrary units of mRNA levels normalized against the expression levels of ef1a amplified from the same template and relative to the average expression observed in eyes of males and females. Asterisks indicate significant difference (p < 0.05) after Student’s t test comparing the expression of male and female tissues.

The transcript levels of RA metabolism enzyme genes (*aldh1a2*, *cyp26a1*, *cyp26b1*, *cyp26c1*) were analyzed for differential expression between male and female medaka (Fig. 3A–D). The *aldh1a2* gene showed high expression levels in the kidney in both sexes. The organ that showed pronounced differential *aldh1a2* expression between the sexes was the gonad, with testis presenting significantly higher expression than ovary. On the contrary, expression levels of *cyp26a1* are extremely high in the ovary, compared to all the other organs in both sexes. The *cyp26b1* and *cyp26c1* genes showed high expression in the gills, but also in brain for *cyp26b1* and in eyes for *cyp26c1*. No significant differences were observed between testis and ovary for both genes. However, a significant difference between male and female was observed for *cyp26c1* in the eye, brain and gills. Taken together, this indicates that *cyp26a1* has a more critical role for sexual development amongst the RA degrading enzymes. Thus, the *cyp26b1* and *cyp26c1* genes were not followed up further.

### Transcript localization of *aldh1a2* and *cyp26a1* and RA responsiveness in adult gonads

To investigate a possible role of RA in male and female gonads of medaka, we attempted to identify the cells that express the RA biosynthesis enzyme gene and the sites of responsiveness to RA in ovary and testis of adult fish.

**Figure 4:**
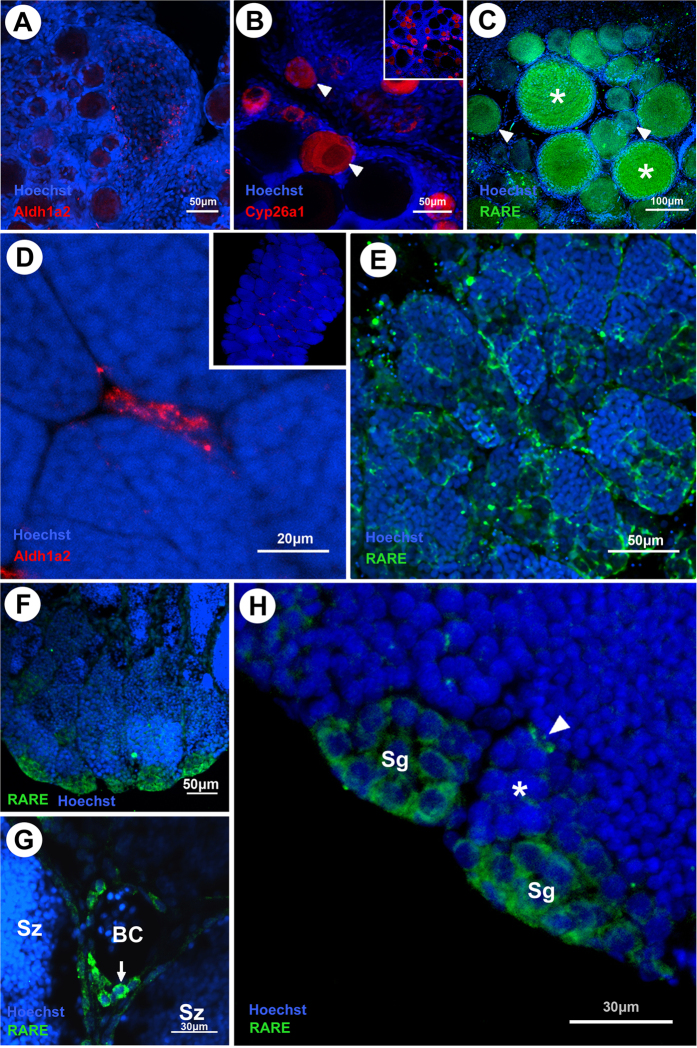
Fluorescent *in situ* hybridization of *aldh1a2*, *cyp26a1* expression and RA responsiveness in adult gonads of medaka. (**A**) *aldh1a2* expression in the parenchyma cells. (**B**) *cyp26a1* expression restricted to early oocytes; strong signal in the prophase I-arrested oocytes (arrowhead). (**C**) Responsiveness to RA present in early oocytes. Note the strong signal in the vitellogenic oocytes (asterisks) (**D**) *aldh1a2* expression in the Leydig cells. (**E**) Responsiveness to RA present in Sertoli and Leydig cells; no germ cells appear to be responsive to RA. (**F**) Responsiveness to retinoic acid at the tip of the germinal epithelium lobe. (**G**) Leydig cells (arrow) close to blood vessels are positive for RARE driven GFP. (**H**) Strong GFP signal in Sertoli cells (arrowhead) and in spermatogonia closer to the tip of the germinal epithelium lobe; Small group of spermatogonia with weak expression (asterisk) more distant from the lobe. BC, blood cells; Sg, spermatogonia; Sz, spermatozoa.

RNA *in situ* hybridization for *aldh1a2* in adult ovary showed no localization in any steroidogenic cells or germ cells (Fig. 4A). *cyp26a1* expression was exclusively seen in the early oocytes and was most prominent in pre-vitellogenic oocytes (Fig. 4B). Analysis of 12XRARE adult females showed that the germ cells are responsive to RA, however only the early oocytes. The signal in early oocytes was detectable already in the pre-vitellogenic stage, increased gradually during vitellogenesis, and vanished completely in post-vitellogenic oocytes (Fig. 4C).

In adult testis, *aldh1a2* was specifically expressed in Leydig cells (Fig. 4D). For *cyp26a1*, no signal was detectable (data not shown). In the 12XRARE transgenic line, strong reporter expression was observed in the somatic cells, in particular in the Sertoli cells at the periphery of the organ, where the spermatogonia containing cysts are located (Fig. 4E). Responsiveness to RA was also seen in germ cells at the tip of the germinal epithelium lobe (Fig. 4F). Leydig cells that were positive for *aldh1a2* also presented responsiveness to retinoic acid (Fig. 4G). At the germ cell level, only the early spermatogonia were GFP positive, and only those Sertoli cells close to GFP positive spermatogonia showed RARE reporter expression (Fig. 4H).

### Effects of modulation of RA signaling in medaka embryos and adult testis

For a functional evaluation of a possible role of RA for meiosis induction, we performed drugs treatments on embryos and adult testis to modulate endogenous RA levels.

Since we did not know how the RA metabolic enzymes genes would respond to exogenously applied agonists or antagonists, medaka embryos were treated 24 hours (from stage 15 to stage 23) with various concentrations of the agonist ATRA (all-*trans*-retinoic acid) and the ALDH inhibitor, DEAB (4-Diethylaminobenzaldehyde), and the expression levels of all RA metabolism genes were quantified.

**Figure 5:**
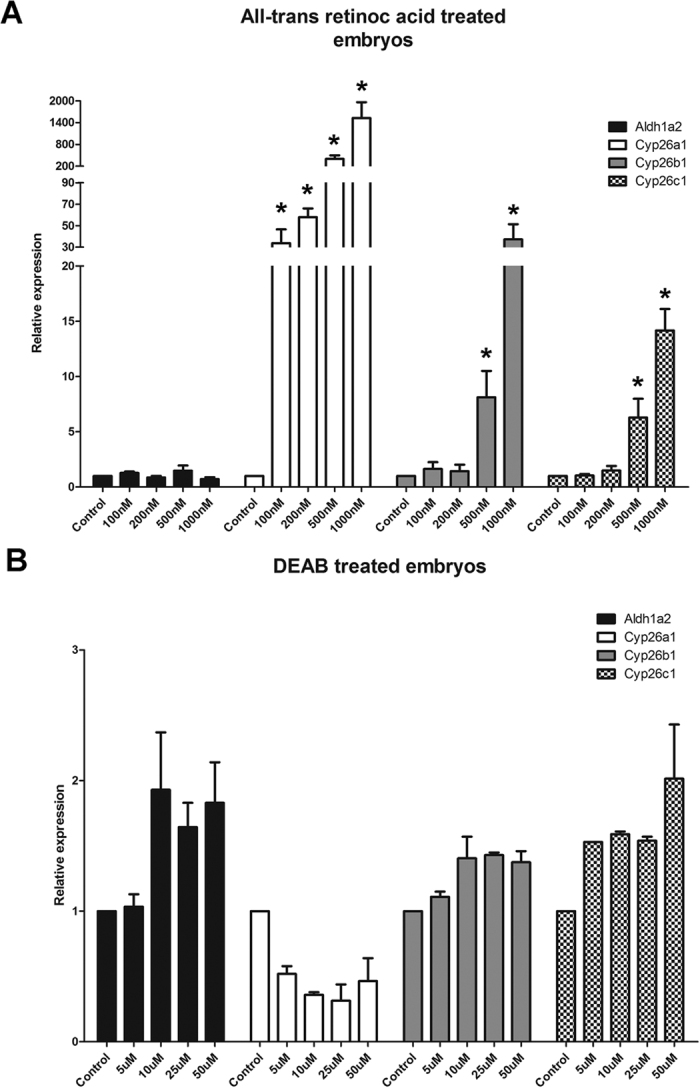
Expression of retinoic acid biosynthesis and degradation enzymes genes in embryos treated with all-trans-retinoic acid and DEAB. Treatment with retinoic acid of embryos from stage 14 onwards for 24 hours. Treatments with retinoic acid (**A**) showed no differences for the *aldh1a2*, but the Cyp26 genes were significantly upregulated at higher concentrations. Treatments with DEAB (**B**) had no strong effects, but at higher concentrations *aldh1a2*, *cyp26b1* and *cyp26c1* were slightly up-regulated, while *cyp26a1* was down-regulated. Asterisks indicate a significant difference (p < 0.05) after Student’s t-test comparing the expression of control and treatment.

In the ATRA treatment no difference in expression levels for *aldh1a2* was observed, but for all *cyp26* genes a profound up-regulation of gene expression proportional to the concentration of the chemical was seen (Fig. 5A). DEAB treatment upregulated *aldh1a2* at higher concentrations, however, this was not statically significant. However, the *cyp26* genes did not respond significantly to DEAB treatments (Fig. 5B).

To analyze how modulation of RA signaling affects the responsiveness to RA, we performed treatments with ATRA, DEAB and Citral (another ALDH inhibitor) chemicals in the 12XRARE transgenic line (Supplementary Figure 3). After treatment with high concentrations of ATRA some new expression domains were observed especially in the cephalic region. We also observed a stronger expression and elongation of the domains anteriorly and posteriorly (Supplementary Figure 3C,H). In the fish treated with DEAB (Supplementary Figure 3D,I) and Citral (Supplementary Figure 3E,J) an almost complete ablation of RA reported expression was observed.

**Figure 6:**
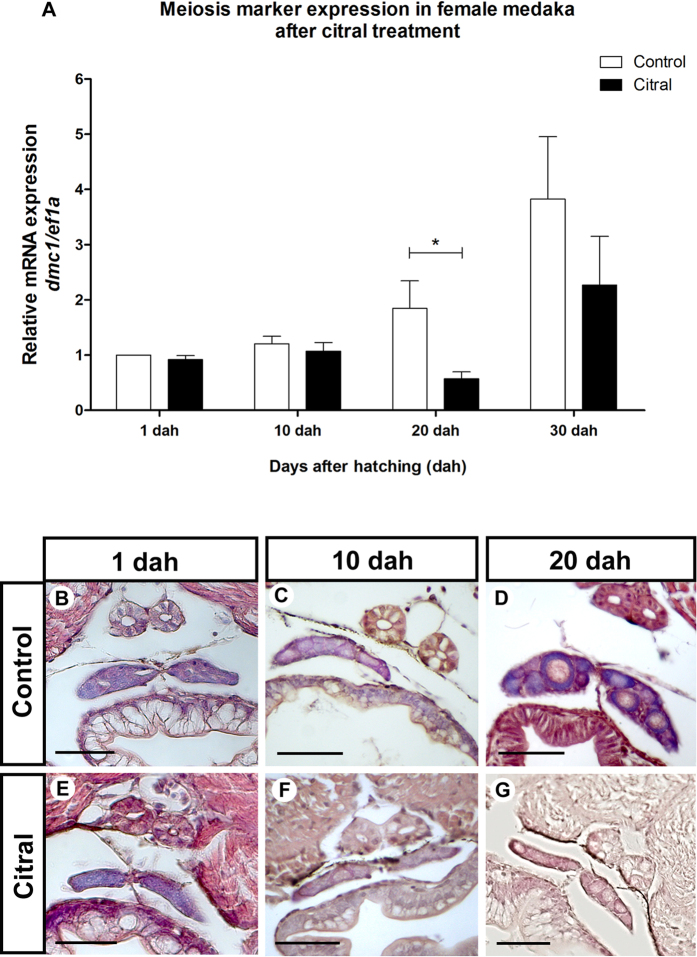
Expression of the meiosis marker *dmc1* and gonad morphology after treatment with Citral in female medakas. Treatment with Citral of female medaka embryos resulting in a lower meiosis marker (*dmc1*) expression (**A**). The morphology of the gonad comparing control (**B**–**D**) and treatment (**E**–**G**) shows only an observable difference at 20 dah. Asterisk indicates a significant difference (p < 0.05) after Student’s t test comparing the expression of male and female gonads. Scale bar = 50 μm.

In mammals, retinoic acid is the main factor that induces the first meiosis in female^15^. We performed long-term treatments of medaka embryos (from stage 29 to 10 days after hatching, dah). Due to a high toxicity of the drugs, only the Citral treatments allowed to maintain the embryos alive until 30 dah. In female embryos, we saw a small delay of expression of *dmc1* (meiosis marker) in all stages, being significantly lower at 20 dah (Fig. 6A). At 30 dah, the expression levels showed no difference to the control animals. By analyzing the morphology of the gonads between the treated and the control, the only differences were observed at 20 dah, where the oocytes are in a more advanced stage of maturation and the size of the organ seems to be bigger in the control (Fig. 6B–G).

**Figure 7:**
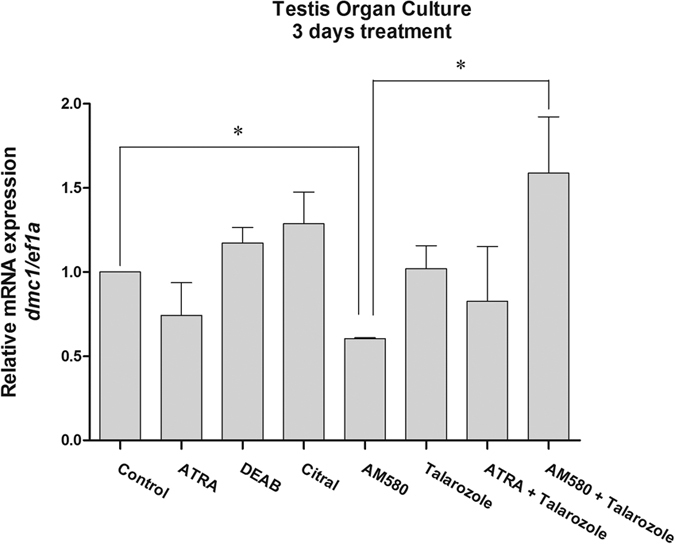
Expression of meiosis marker *dmc1* gene after treatment of testis explant culture. Treatment with AM580 resulting in downregulation of *dmc1* after 3 days of treatment. A small rescue is observed be treating the testis with both AM580 and Talarozole (CYP26 inhibitor). Asterisks indicate a significant difference (p < 0.05) after Student’s t test comparing the expression of male and female gonads.

To assess a possible role of RA in the adult gonad, we performed treatments of testis explants (Fig. 7). After three days of treatment no effect in expression levels of *dmc1* for those drugs that inhibit RA synthesis (Citral and DEAB) was recorded. Treatments with ATRA, AM580 (RA receptor α agonist) and Talarozole (CYP26s inhibitor) are expected to stimulate the RA pathway. For AM580, we found a significant decrease in the expression of *dmc1*, while the other two drugs had no effect. Interestingly, combined treatment of testis explants with both AM580 and Talarozole led to a 3-fold significant upregulation of *dmc1* in comparison to the explants treated with AM580 alone.

## Discussion

The cognate role of RA in germ cells of mammals is to induce the cells to enter into the first meiosis^9^,^15^,^16^. However, involvement of RA in germ cell fate and meiosis entry in fish is not well known. In medaka, some information on the expression pattern of RA biosynthesis enzyme genes during development has been reported, but the gonad had not been studied^22^,^25^. Medaka is a well suited model species to study sex determination^26^. As a first step towards an understanding of the role of RA in sex determination and sexual development, we analyzed the RA metabolism gene expression patterns during development and in gonads of the adult fish. To obtain evidence for a functional role, we also monitored RA transcriptional control activity by using a RA responsive reporter gene expression system and did treatments with RA agonists and antagonists.

We observed that often the source of RA production and its site of action are uncoupled, as it is typical for a diffusible morphogen^20^. For instance in the retina of medaka *aldh1a2* is expressed equally in the ventral and dorsal retina, like described in zebrafish, but only the ventral retina seems to be strongly responsive to the RA^27^,^28^. In the gonad at embryonic stage 33, RA is synthesized, but RA responsiveness is exclusively in the nephric duct. This pattern was already described in zebrafish where during early kidney formation the RA signal is required for patterning the rostral/caudal domain of the renal progenitor cell, however the retinoic acid does not come from the pronephros itself but from the paraxial mesoderm adjacent to the kidney^29^. In mammals, the *aldh1a2* gene is not expressed in the developing gonad, but the mesonephros is the source of RA^15^,^16^.

Taking these results together, it is likely that in teleosts, different from mammals, RA is derived only from the gonad itself, having no additional RA originated from the mesonephros, like what was proposed by Rodriguez-Mari *et al.*^20^. They suggest that this model is consistent with the fact that the mesonephros in zebrafish lies distant from the gonad and does not contact the gonad during the critical time of sex determination. Our data also confirm the hypothesis of an effect of RA in kidney development, despite its absence of production in the nephric ducts in fish^29^,^30^. However the conclusion that only teleosts then need such an early expression of *aldh1a2* in the gonad, does not hold true, since chicken also presents this pattern^18^. Thus, it appears that expression of *aldh1a2* in the mesonephros, but not in the early gonad, is a characteristic that is not conserved in all vertebrates.

The biological effects of RA are mainly mediated by all-trans-RA binding to nuclear RARs. RARs bind to their DNA through response elements RAREs, which depending of the presence or absence of the ligand, regulate the transcription of multiple target genes^31^. *In-silico* promoter analyzes of zebrafish *cyp26a1* showed the presence of two canonical RARE that are also conserved in mouse^32^. The promoter region of medaka *cyp26a1* also contains these two canonical RAREs (Supplementary Figure 4). Certain tissues express *cyp26a1* in an apparent RA-independent manner^33^, however, it was demonstrated that endogenous RA is involved in controlling the expression of *cyp26a1* in cells within or adjacent to the presumptive hindbrain during gastrulation of zebrafish^34^. Treatment of embryos, tissues, and cells with exogenous RA strongly induces expression of *cyp26a1* in a time and concentration-dependent manner^34^,^35^,^36^. This was also observed in the all-trans-RA treated medaka embryos, where higher concentrations of RA correlated with higher expression of *cyp26a1*. This observation was also true for *cyp26b1* and *cyp26c1*, but only with the highest concentrations. However, a strong up-regulation of *cyp26a1* was already observed at the lower concentrations of RA, meaning that the *cyp26a1* gene, which is preferentially expressed in female gonads, is most sensitive to RA-treatment induced regulation. Treatment with DEAB showed expression difference only for *cyp26a1*, where the gene was significantly down-regulated. It appears that the level of *cyp26a1* is responding directly and most sensitive to the levels of exogenous and endogenous RA.

During gonad development of medaka, entry into meiosis occurs first in females (around hatching) and later in males (around 20 days after hatching)^37^. We detected responsiveness to RA in the germ cells of medaka around stage 30. Notably, this is at least 6 days before the first sign of meiosis entry in females can be observed. The window of RA expression coincidently matches with the critical period where sex can be reverted in female embryos to develop as fertile XX males^38^. This period is characterized by expression of the male sex determining gene *dmrt1bY* and is then followed by a mitotic arrest of the PCGs in the male gonad^39^, and by the change of type I to type II proliferation of germ cells in female gonads^40^. However, at the hatching stage, which is characterized by meiosis entry in females, we did not detect any RA responsiveness of the reporter construct.

Long-term treatment with Citral showed that entry of meiosis can be slightly delayed, but effects of inhibition of RA synthesis become only evident at later stages (20 dah), long after the first entry into meiosis. Unexpectedly, we noticed an early responsiveness to RA in the 12XRARE transgenic line considerably earlier than the first meiosis in females. As this responsiveness is seen in both sexes, it is unlikely that it has any function related to sex determination. One possibility is that it may be a general feature of PGC development. On the other hand, expression of other genes at this time has been reported, but these show sex specificity^2^,^37^,^40^,^41^,^42^. Future experiments employing conditional knockout of these genes might help to resolve the question on the functional meaning or these expression. Recent work in Tilapia showed that treatments with an inhibitor of RA synthesis or disruption of *aldh1a2* leads to a delay in entry of meiosis, similar to our results with medaka^21^. However, it cannot be excluded that the drugs cause an unspecific delay of development of the hatchlings.

In adult testis of mouse, expression of RA-synthesis genes was found in the germ cells, Sertoli cells and Leydig cells^43^. In zebrafish, no gonadal expression of *aldh1a3* was observed and *aldh1a2* was detected in the undifferentiated gonad and in the adult gonad, in Sertoli cells for testis and in the somatic interstitial cells for the ovary. Medaka *aldh1a2* is also expressed in the adult gonad of both sexes, where higher expression occurs in testis than in ovary. This suggests that a bias in levels of RA is important still in the adult gonad. In mammals, a difference between male and female was also observed. This is in agreement with the findings in fish. Medaka *aldh1a2* is the only member of the *aldh1* gene family, which combines the roles of *aldh1a2* and *aldh1a3* of zebrafish^22^. Thus, gene expression is restricted to this single paralog in medaka and may explain the high differences of *aldh1* expression between male and female. One may speculate that if there is an early testis differentiation specific function of *aldh1a2* in medaka, which might be maintained in the adult, like for most male sex genes^2^.

In mouse, only *cyp26b1* regulates the timing of meiosis onset. The undifferentiated gonads (11.5 dpc) express *cyp26b1* at low levels in both male and female. After expression of *Sry* in male gonad (12.5) levels of *cyp26b1* are increased in the testis, and at the same period the levels of *cyp26b1* are reduced in the ovary. In females, RA acts in the early ovary, leading the cells into meiosis and oocyte development by activating *stra8*^17^,^44^. In zebrafish, the gonads of both sexes pass through a transitional stage acquiring a juvenile ovary-like gonad with immature oocytes. In females, these oocytes continue to develop and reinforce the development of mature ovaries, but in males, oocytes die by apoptosis and the gonads then become testis^45^. Hence, no sexual dimorphic onset of meiosis can be recognized.

In zebrafish, Cyp26a1 is the main RA-degrading enzyme in the adult gonad^20^, instead of Cyp26b1 as in mammals. The function of meiosis blocking for *cyp26a1* in zebrafish appeared to be conserved, since this gene was found to be only expressed in those somatic cells that are not surrounding meiotic cells. In females, the expression is restricted to the oocytes arrested in meiosis. Our data in medaka show that *cyp26a1* has higher expression levels in ovary, where it specifically localizes to the early oocytes, in agreement with the findings in zebrafish. However, our data from the 12XRARE transgenic reporter line showed that RA is acting also in oocytes that already have resumed meiosis. Thus, *cyp26a1* is up-regulated in oocytes that entered prophase-I meiotic arrest, and is downregulated when meiosis is resumed^20^. These data are in agreement with the hypothesis that *cyp26a1* acts as a meiosis inhibitor.

However, a role of *cyp26a1* in meiosis arrest may be sexually dimorphic in zebrafish and medaka: in the testis Sertoli cells protect spermatogonia to enter meiosis, and in the ovary *cyp26a1* is expressed in the early oocytes (late stage 1B) that are arrested in diplotene^20^. This fact together with the high levels of *aldh1a2* in testis leads us to infer that there is a sex specific role for RA in male, in addition to meiosis induction. On the other hand in the ovary it has to be strongly repressed because it would boost meiosis entry of germ cells. Together with the fact that *aldh1a2* has a specific Leydig cell expression, we hypothesize that RA may be not only related to meiosis, but also to the maintenance of the male gonad identity. In the adult testis of medaka, interestingly, we also found sexually dimorphic RA responsiveness. In males, the response to RA is observed in Leydig cells, type A spermatogonia and Sertoli cells close to the RA-responsive spermatogonia. The tip of the germinal epithelium lobe of medaka testis is the region to which type A spermatogonia are restricted^41^, and those cells are pre-meiotic cells^46^. This may indicate that RA is acting in the germ cells by inducing gametogenesis and consequently meiosis.

Experiments in mouse show that RA plays an important role in initiation of spermatogenesis and in proliferation and differentiation of Sertoli cells^47^. In testis explants culture, we find evidence that RA may interfere with meiosis rather than initiating it. A significant decrease of meiosis marker was observed after treatment with the RARα agonist rather than up-regulation of the meiosis marker. This explanation would be supported by the observation in mammals that the entry of meiosis occurs only after 9 days of the induction by RA. After this induction, the undifferentiated spermatogonia go into differentiation, followed by 5 cycles of division and just then undergoes meiosis. In the case of medaka, differentiating spermatogonia within a cyst are connected to each other through cytoplasmic bridges and clonally divide 9–10 times before entering meiosis^48^. In our experiments, we provided a 3-day treatment, meaning it is likely that those germ cells being induced by RA still did not enter meiosis. However, this does not explain the down-regulation of the meiosis markers after treatment.

The counteraction of the RA metabolism enzymes puts a note of caution to all treatment experiments. Considering that RA signaling is a complex and delicate pathway, it could mean that treatments with antagonists and agonists might not reflect the physiological situation and allow us to infer effects of endogenous RA. The down-regulation of meiosis marker in our organ culture experiments, and a not clear induction of meiosis by RA treatments, can be related to the strong up-regulation of the degrading enzymes after induction be exogenous RA.

## Conclusions

Our findings allow us to suggest a complementary hypothesis for the control of meiosis entry through RA, adding to what was proposed for zebrafish in the adult gonad. In female medaka, like in zebrafish, it is conceivable that RA has the function to release the oocytes that were arrested in meiosis during oocyte maturation^49^,^50^. This is partly because of the early signal to RA responsiveness in the later 1B oocytes. In males, on other hand, entry of meiosis is probably related to a direct action of RA in the germ cells. However, an indirect role of RA in meiosis and gametogenesis cannot be excluded, since Sertoli cells and Leydig cells also present responsiveness in adult testis. This is in line with the hypothesis proposed by McLean *et al.*^51^, that RA induces one or several so far unidentified factors in Sertoli cells that are secreted and initiate meiosis in the germ cells.

In the embryo, we find no evidence that RA is essential for the first entry of meiosis in medaka, contradicting what is known in mammals and other vertebrates. One possible explanation may come from the fact that the majority of teleosts, from which we have information so far, do not have a *stra8* orthologue. In Tilapia, it was shown that RA is important, but not necessary, for the entry of meiosis^21^. On the other hand in catfish, it seems that this process is similar to mammals, as this species possesses a *stra8* gene^52^,^53^. Since both medaka and Tilapia do not have *stra8*, the commonality of both fish lacking this important mediator of RA action is in line with the absence of an effect during the sex determination period. However, many other explanations for a difference in the mechanisms that regulate the entry into the first meiosis exist and further experiments with an unbiased approach should help to find the molecules that are instrumental in this process.

## Methods

### Animals

Medaka (*Oryzias latipes*) fish belongs to the class Actinopterygii, order Beloniformes and the family Adrianichthyidae. It is tiny fresh water species, which is widely used as a laboratory organism for many biological questions and in biomedical research^26^.

All experiments were performed with Carbio strain of medaka. The animals were kept under standard photoperiod cycle of 14 hr/10 hr light/dark at 26 °C (±1 °C). Eggs were collected 1–2 hr after starting the light cycle and raised at 26 °C in Danieau’s medium (17.4 mM NaCl, 0.21 mM KCl, 0.12 mM MgSO_4_, 0.18 mM Ca(NO_3_)2, 1.5 mM Hepes, pH 7.2). The stages of development were identified according to Iwamatsu^54^.

All animals were kept and sampled in accordance with the applicable EU and national German legislation governing animal experimentation, in particular all experimental protocols were approved through an authorization (568/300-1870/13) of the Veterinary Office of the District Government of Lower Franconia, Germany, in accordance with the German Animal Protection Law (TierSchG).

### Production of 12XRARE reporter transgenic fish and imaging analyses

To make a transgenic RA reporter, we used the 12XRARE reporter vector (12XRARE-ef1a:gfp), which has a concatenation of 12 direct repeat 5 (DR5) retinoic acid response element (RARE) sites. As described previously, the RARE sites were placed upstream of an elongation factor-1 alpha (ef1a) minimal promoter in a vector containing *egfp* flanked by adeno-associated viral inverted terminal repeat elements and I-SceI sites^27^. The plasmid was injected at a concentration of 100 ng/μl together with the *I-SceI* meganuclease enzyme (0.5 unit/μL in 0,5 X *I-SceI* buffer), through the chorion into the cytoplasm of the one-cell stage embryo^55^. Embryos were kept at 28.5 °C until hatching. GFP- positive G_0_ fish were mated to each other and the offspring were again sorted for fluorescence.

We generated double transgenic reporter lines by crossing 12XRARE-ef1::gfp with Vasa::H2B::mCherry and sox9b::mCherry^2^ fish to identify the germ cells and the somatic cells, respectively. The Vasa::H2B::mCherry medaka transgenic line was kindly supplied by Dr. Alexander Froschauer from the Zoology and Developmental Biology, Technical University of Dresden.

For imaging embryos, hatchlings or tissues were mounted with 1–2% low melting temperature agarose. Confocal pictures and image stacks were acquired using a Nikon C1 (eclipse Ti) confocal laser scanning microscope and the NIS elements AR software.

### *In vivo* all-trans retinoic acid, Citral and Diethylaminobenzaldehyde (DEAB) treatments

All-*trans*-retinoic acid (ATRA; SIGMA-ALDRICH) and DEAB (SIGMA-ALDRICH) were diluted in DMSO as 1 mM and 10 mM stocks, respectively, and stored at −20 °C in the dark. Serial dilutions for RA (100 nM, 200 nM, 500 nM and 1000 nM) and DEAB (5 μM, 10 μM, 25 μM and 50 μM) were made in Danieau’s solution just prior to use. Medaka embryos were collected and raised in Danieau’s medium. From the dome stage (stage 14) onwards, the embryos were kept in the dark for 24 hours (stage 22). The concentration range is based on reports of similar treatments in other fish^56^,^57^.

To investigate an effect on meiosis entry, we made long term treatments from stage 29 until 10 days after hatching (dah) of Citral (1 μM; SIGMA-ALDRICH), and the medium was changed every 2 days. Specimens were collected at 1 dah, 5 dah, 10 dah, 20 dah, 30 dah, 40 dah and 50 dah and genotyped for sex by PCR amplification of the Y-linked male determining gene *dmrt1bY* using genomic DNA as template. For genomic DNA extraction, a piece of tissue was incubated for 1 hour at 95 °C in 100 μL of a basic buffer solution (25 mM NaOH, 0,2 mM EDTA, pH = 12) with gentle shaking. The solution was cooled down on ice, 100 μL of neutralization solution (40 mM Tris-HCl pH = 5.0) added and vortexed. 2 μL of the total volume was used in a 25 μL PCR reaction. The PCR products were resolved in 1% agarose gels.

### Testis organ culture

For experiments on meiosis induction and inhibition in adult testis, we performed *ex vivo* organ cultures as developed for zebrafish testis^58^. Medaka gonads during the development start as two separated tissues and later on fuse together into one^42^. The gonads of adult male were dissected, and the fused testes were separated in two parts; one was used as experimental control and the other for treatments. Considering that the cell cycle time of spermatogonia in fish is approximately 30 hours^59^ we used an incubation period of 3 days.

Triplicates of testis samples were incubated either with *all-trans*-retinoic acid (ATRA, 1 μM), DEAB (2, 5 μM), Citral (1 mM), CYP26 inhibitor Talarazole (1 μM) and RAR agonist AM580 (10 nM), in presence or absent of Talarazole. After incubation the testes were used for gene expression studies.

### Real time quantitative RT-PCR

Tissues of adult males and females and whole embryos of different developmental stages from the Carbio strain were collected. Total RNA was extracted from 3 pools of adult fish tissues (n = 4) and whole embryos (n = 20) using the TRIZOL reagent (INVITROGEN) according to the supplier’s recommendation. After DNase treatment, reverse transcription was done with 2 μg total RNA using RevertAid First Strand Synthesis kit (THERMO SCIENTIFIC) and random primers. Real-time quantitative PCR was carried out with SYBR Green reagent and amplifications were detected with a *Mastercycler ep realplex* (EPPENDORF). All results are averages of at least three independent RT reactions from three independent RNA preparations. Transcript levels of the target genes were normalized against the medaka elongation factor-1 alpha (*ef1a*) gene. The ΔCt values presented as means ± standard error of the mean (SEM), were analyzed by one-way ANOVA, Tukey’s and Student’s t-test. A significance level of P < 0.05 was used for all tests.

### *In situ* hybridization

To generate riboprobes, cDNA fragments corresponding to the 3′ untranslated region (UTR) and the open reading frame (ORF) of *aldh1a2* and *cyp26a1* were PCR amplified from cDNA of adult medaka testis and ovary respectively (for primers sequences see Supplementary Table 1). The amplified fragments were subcloned and used as template for *in vitro* transcription. Whole-mount RNA *in situ* hybridization was performed as described^60^. Stained embryos were dissected and mounted in glycerol for photography. Staining time was individually adjusted for each probe to get the best signal and does not reflect the endogenous transcript expression level. For a more sensitive fluorescence *in situ* hybridization detection, we used the HNPP Fluorescent Detection Set (ROCHE) according to the manufacturer’s instructions.

*In situ* hybridization on sections was carried out on 5 μm-thick paraffin sections. After paraffin removal the sections were rehydrated and treated with proteinase K (10 μg/mL; SIGMA-ALDRICH) at room temperature for 10 min. Hybridization with 400 ng riboprobe per milliliter was carried out in hybridization solution (5x standard sodium citrate, 50% deionized formamide, 50 μg/ml heparin, 0,1% Tween-20, 50 mg/ml yeast tRNA) at 70 °C overnight. The slides were then washed in 5x and 0,5x standard sodium citrate in 20% formamide at 60 °C, each 3 times for 20 min, and blocking was performed with 1% Blocking Reagent (ROCHE) at room temperature for 1 h. Subsequently, the slides were incubated with alkaline phosphatase-conjugated anti-DIG antibody (ROCHE) diluted 1:2000 in blocking solution at 4 °C overnight. Color development was conducted by incubating the sections in Nitroblue tetrazolium/5-bromo-4-chloro-3-indolylphosphate (ROCHE) or with the HNPP Fluorescent Detection Set (ROCHE) in the dark. After air-drying, the slides were mounted in glycerol for photography and micrographs were taken with a ZEISS Axiophot microscope.

### Light microscopy

Larvae and gonads from adult fish were dissected and fixed in 4% paraformaldehyde solution for 24 h at 4 °C, subsequently dehydrated, embedded in paraffin, and then serially sectioned at 5 μm thickness. The sections were counterstained with hematoxylin & eosin (HE).

## Additional Information

**How to cite this article**: Adolfi, M. C. *et al.* Retinoic acid and meiosis induction in adult versus embryonic gonads of medaka. *Sci. Rep.* **6**, 34281; doi: 10.1038/srep34281 (2016).

## Supplementary Material

Supplementary Information
