# Supplementary material for: Retinoic acid and meiosis induction in adult versus embryonic gonads of medaka
Source: Sci Rep. 2016 Sep 28;6:34281. doi: 10.1038/srep34281 (PMC5039705; doi:10.1038/srep34281)
Supplement: Supplementary Information [file srep34281-s1.pdf]

Supplementary information

Retinoic Acid and Meiosis Induction in Adult versus Embryonic Gonads of Medaka

Mateus C. Adolfi[1], Amaury Herpin[1,2], Martina Regensburger[1], Jacopo Sacquegno[1], Joshua S. Waxman[3] and Manfred Schartl[1,4]

1 -University of Wuerzburg, Physiological Chemistry, Biocenter, Am Hubland, D-97074 Wuerzburg, Germany.
2 - INRA, UR1037, Fish Physiology and Genomics, Rennes F-35000, France.
3 - The Heart Institute, Molecular Cardiovascular Biology and Developmental Biology Divisions, Cincinnati Children's Hospital Medical Center, Cincinnati, Ohio, USA.
4- Comprehensive Cancer Center Mainfranken, University Clinic Würzburg, Josef Schneider Straße 6, 97074 Wuerzburg, Germany and Texas Institute for Advanced Study and Department of Biology, Texas A&M University, College Station, Texas 77843, USA

SUPPLEMENTARY TABLE

Primer	Sequence	Purpose
Aldh1a2_RT_F	5’-GCCTTCTCTTTGGGCTCAGT-3’	qRT-PCR
Aldh1a2_RT_R	5'-GGAAATGCGGTGTAGTGGTC-3’	qRT-PCR
Cyp26a1_RT_F	5'-AGAGATGAAGCGGCTGATGT-3’	qRT-PCR
Cyp26a1_RT_R	5'-TCTTCTTGCTGCTGTCGATG-3’	qRT-PCR
Cyp26b1_RT_F	5'-ATCCGAGCACGAGACATCTT-3’	qRT-PCR
Cyp26b1_RT_R	5'-GGATGTTGGAGGAGTTGCAT-3’	qRT-PCR
Cyp26c1_RT_F	5'-CAGAGGGGCTTGACTTTGAC-3’	qRT-PCR
Cyp26c1_RT_R	5'-CAGCCTTTGGGAATCTGGTA-3’	qRT-PCR
Dmc1-RT-F	5’-GTTTCCAGACGGCCTTTGAG-3’	qRT-PCR
Dmc1-RT-R	5’-CGATGTCTCTCAGCCTGTCT-3'	qRT-PCR
Ef1a-RT-F	5'-GCCCCTGGACACAGAGACTTCATCA-3’	qRT-PCR
Ef1a-RT-R	5’-AAGGGGGCTCGGTGGAGTCCAT-3'	qRT-PCR
Aldh1a2_Probe_F	5’-CAGAGTGGAATCCAGGAAGG-3'	*in situ* hybridization probe
Aldh1a2_Probe_R	5’-GGAAATGCGGTGTAGTGGTC-3'	*in situ* hybridization probe
Cyp26a1_Probe_F	5’- CTTTCAGCTCAATGGCTTCC -3'	*in situ* hybridization probe
Cyp26a1_Probe_R	5’-CAAACGTCATGAGCAGCCTA-3'	*in situ* hybridization probe

Supplementary Table 1 –Sequence of primers used in the present study

SUPPLEMENTARY FIGURES

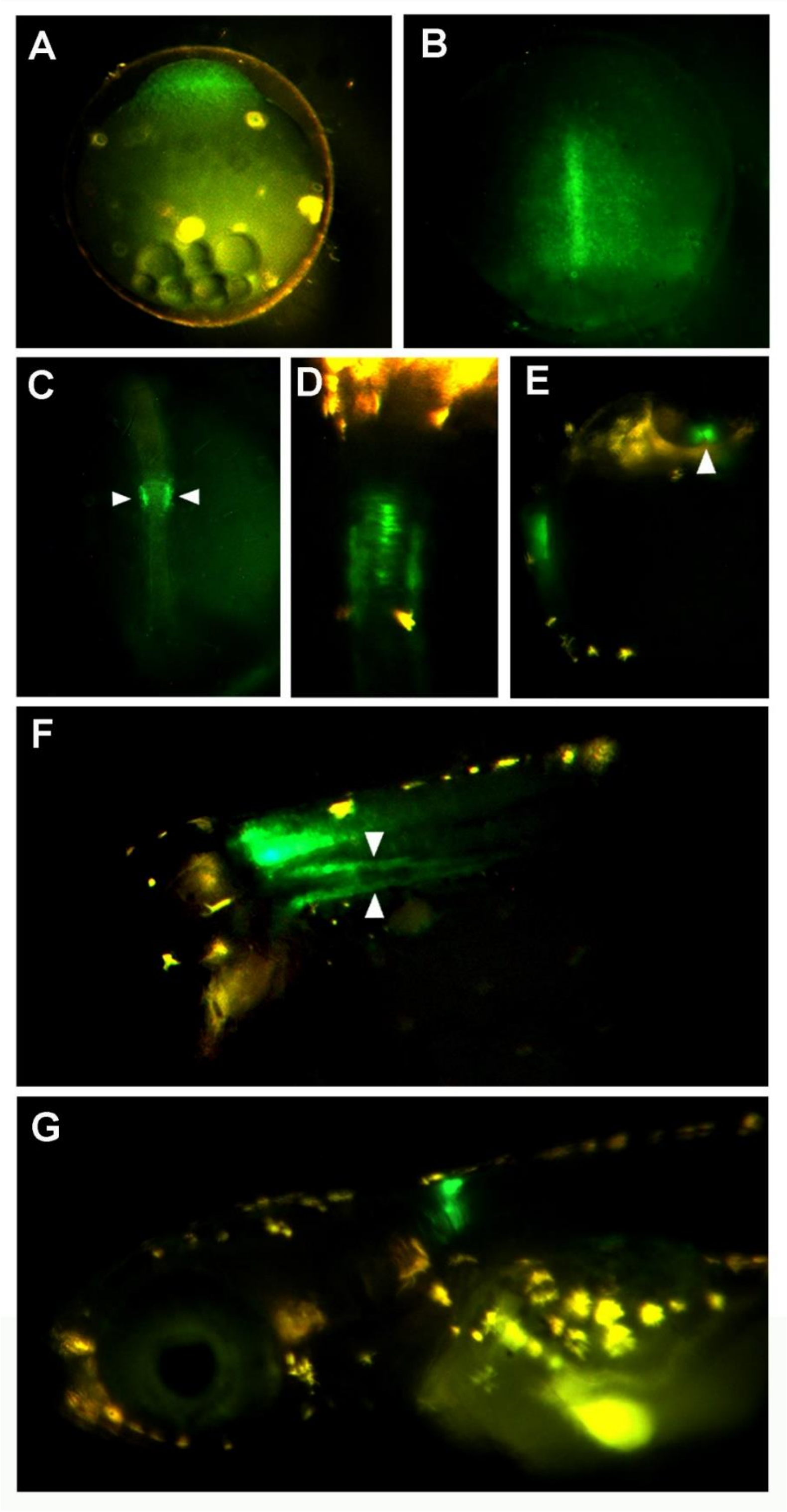

Supplementary Figure 1. Spatial and temporal expression patterns of 12X Retinoc Acid Responsive Element (RARE) transgenic line during medaka development. (A) Early blastula stage with ubiquitous RA-resposiveness. (B) Late gastrula stage with ubiquitous responsiveness on the embryonic shild. (C) Stage 18 with responsiveness on the lateral plate mesoderm (arrow head). (D) Stage 23 (12 somite stage) responsiveness in the somites (until 5-6), neural tube and notochord. (E) Stage 27 showing responsiveness in the retina, strongly at the ventral part (arrow head). (F) Stage 33 with pronephros responsiveness clearly recognizable (arrow heads). (G) Hatching stage, with responsiveness of the hindbrain being more prominent compared to other tissues.

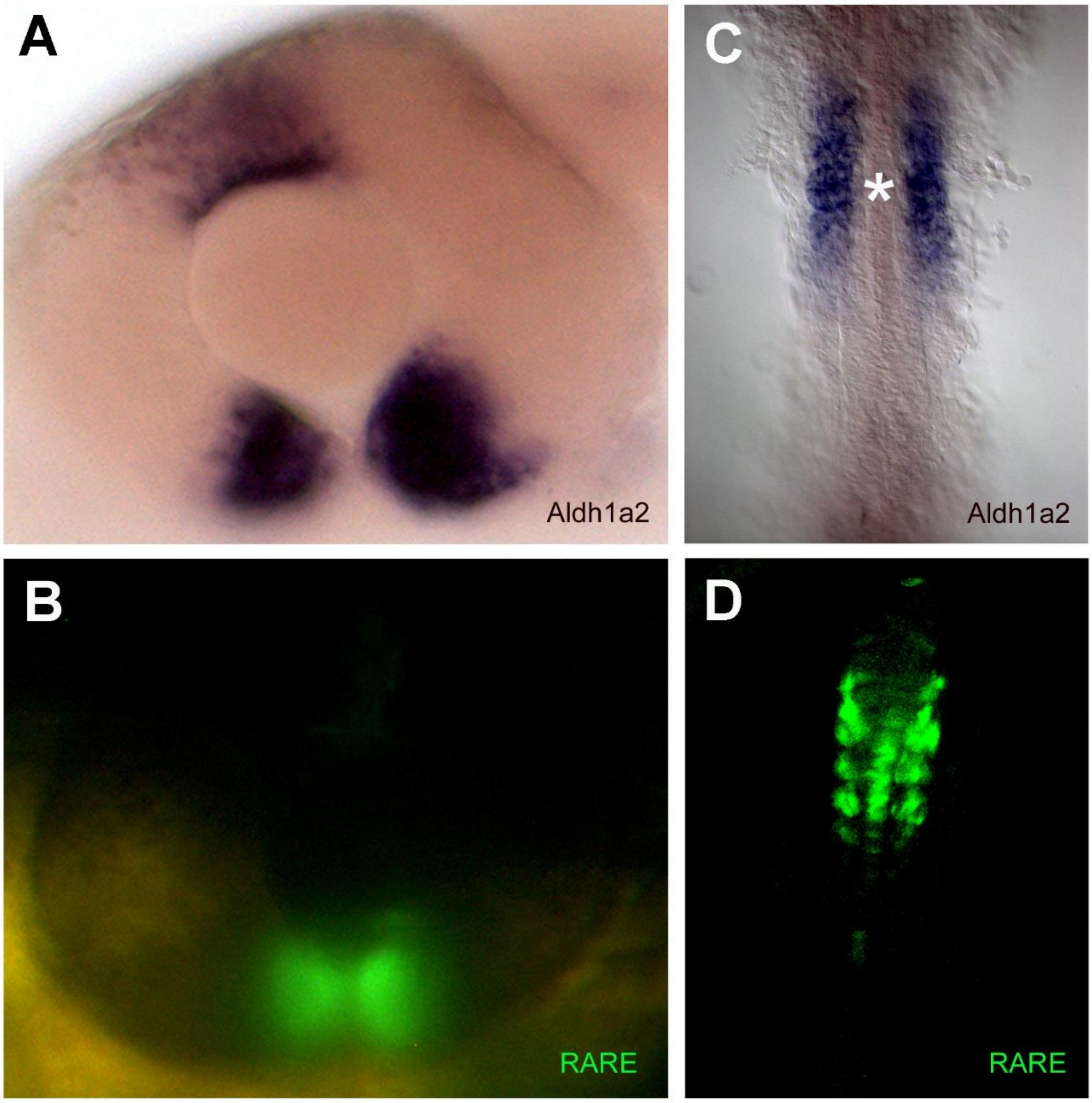

Supplementary Figure 2. Comparison between *aldh1a2* expression and responsiveness to retinoic acid in medaka embryo. (A) *in situ* hybridization for *alh1a2* with ventral and dorsal expression domains in the retina. (B) RA responsiveness in the ventral retina. (C) *in situ* hybridization for *alh1a2* showing expression in the first four somites. (D) GFP expression in the first four somites and the neural tube of a *tg(12xRARE:GFP)* embryo. The asterisk indicates the region where RA action is not restricted to its source of synthesis.

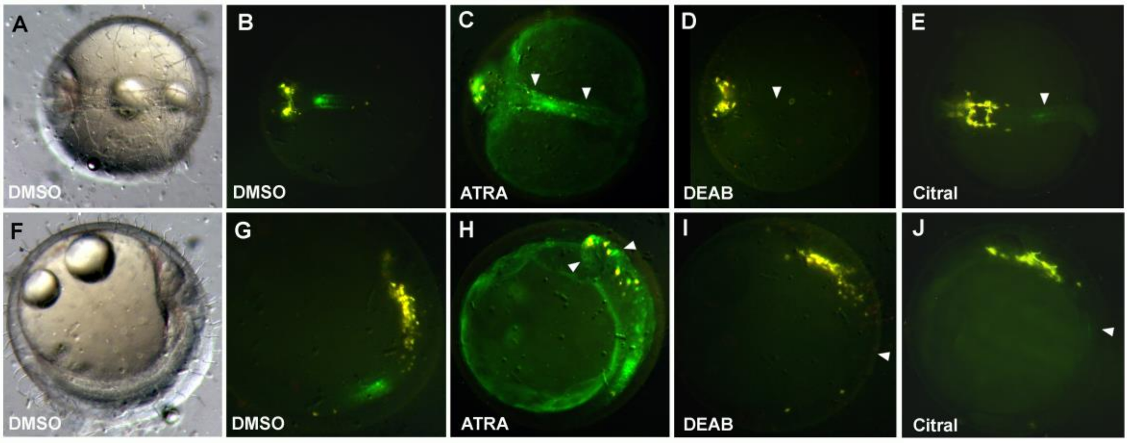

Supplementary Figure 3. 12XRARE transgenic signal after ATRA, DEAB and Citral. Treatment of embryos with 100 nM ATRA (C and H) starting at stage 14 induces transgene expression throughout the neural tube and neural retina by stage 23 (arrow head) compared to the DMSO-treated control (A, B, F and G). Treatment of embryos with 5 µM DEAB (D and I) and 1 µM Citral (E and J), both RA synthase inhibitors, at the same stage significantly reduces transgene signal in the neural tube (arrow head) compared to DMSO-treated control.

```
-2392 ACAGTGATGCACGTGAACCACGATGAGGTTTGGTGGCTGTTTCTGTACCAAAGTCGCTGATTTCACCTGTCAGATGTATCAAAACAATCTGTGCTTGACT -2293
                                                                                                  -2207  -2200
-2292 CCCAAAATAGCATCGTGTCACCGCGTCTACGGCTCCCATTGAACTCAGACGTTCCATTGACTTTTCTTTTTTTTGACGGCTTAAGCTGAACTCTGAACCA -2193
-2192 GACCTTGTCTGTTATCAGTGGATATCACCAAACTTTAACTTAAATCTGAATCAAAACCCAAGAAAACCTTTTACACGAGGAAATGAAATGAAGCCCACTG -2093
-2092 GTCCAGGTCTGTTTTTTGGTCACACCTCTGACTAGGACAGGAGTCCACTAAAGTCTCTAATGGAAATCAAATCCGTCTTTCTCTAAGTCACATAAAAGTC -1993
-1992 CTTCCATAAGTAAATTTAAAAAAAAACTAAATTTCAAATGACTTTTATTCATAACACTGAAGATAAAGTTCATTTAAATGCCTTTTTTCCACACCTAAGG -1893
-1892 TCACAGGGCAAACGAATAATGAACCAAAATGTTTTATCTATAAACTTAAAGATGGAGCAGATCAGGACTCCACCCTGACAGAACAAGATAGCCTAAACAG -1793
-1792 ACGAGTTTTCAGTTTTTATTTGAATTGTGGAAAACAGATAGTGTTTCGGAGATGTAGACAGGATCCATACCTGGGGGGCAGACTGCTCTCTGTCCAATGG -1693
-1692 AAGTGAGACAAGCAGAGGTGGGGAGGGGGGTGGAGTTATGGAGATAGCCAGACGAGATCAGAGCGAGGCTGTGGACGGTCTTGAAAGTAAAAACACGTCT -1593
-1592 GTGACATTTGAACGGAGGTGATGCTAAAACTGGACCAGGATAAGCGCTTCACTCTTCGTTCATACTTGGTGACGGTGGAGGCCTGGCTGCCAGTTCCCAC -1493
-1492 CTACGGTCCCTCTGACCAACACCAGGATCATTCATGCACAATCATGATGGTAGGCAGTAGCGTTAAAGGTCTTGCCCAAGGGCCCTCACTGGGTGTTACC -1393
-1392 TGACTGGTGTTCTGCTCACCAATTCAATTTCTTGCTTGTTCATTGCCCGCCACGAATTGAACCCTGCATGTCAGTCCTTCCCCTTACCAACCGAGCTACC -1293
-1292 CAGCCGCTCATGTGGTAGTGTGACATGTGATAGTAGGACAGAGCTCTGAACACCAGAGCTGACTAAGAGAGGATGGACAGATTTGAACTGCTGCTGAAAG -1193
-1192 CAGATTAGGCATGGATGGAGTCAAAGGCCTCGCTCCGACCACGCAGGATGAACCCCCGACGGCGAGGAGGAAGGTCAGTAGTTTGGATTGAAGCAGTCAG -1093
-1092 TCTCTGTGAAAGGGAGAGGAGGAAACCCCCGTTTATTCAAATGATTTCATAAAAGACATTTTTCAATGGAAAAATGTTGAGATCATTGGAACCAGCAGCA  -993
-992  GTTTTAGAAATGAGAGAGGAGAGAGTCGTGAGGAGAGATGCCAAAGAGGAGGTGGGGGCTTTGAGAGCAGCAGAGGGAGGGAGATCCTCCTGATGGTTCC  -893
-892  TGTTTGATGAGACATGCAGTCACTTTAGTGACAAATGAGAAGAAATAAAAGGTTTAAAAAATAAAGCGTGTAAAAGCACAGCCAGCCTGTAACTTCAGAC  -793
-792  TGTCTGGCTCCGCAGTGCCGCGCGTGCGCGCGCACCCACATACCTGATTGAATGACGACAGCGTCAGGGATCCAAACACAACAGATCCACGCGCAAAGTG  -693
-692  GGGATGTCGTGGTGCACTGCTGCCCCCCCCCCCCCCCTGTTTGCGTGCAGTGGCGTGGCCCGGGGATAACCCTGCGTGCGTTTGCGCGCGCGCCCTTGCAT  -593
-592  GCCTCATCTGACCCAAGTTTGAACCCGGACAGAGATATCATTATAGCCGCCGCGCGGCTATGTGGAGGGTAAAGGGGGTTCTTGGGGGCCCGGTGGTCCG  -493
-492  GGCGTGCCCGGGGCCCGGCGTGCGTGCCAATGGTGCGTTCAGGGGCCCCGAAGAGCCTCTTTACCATTTAGTAAAAGCCGCGCTAAGCCCGTTTTGCTTG  -393
-392  TGAATGCAGGCCGTCGCCCTGCAGGCATTTGTGCTGTTCCTTAAGTGGCTCTTGGCGCGGTCATGAGGGTAACAAAGGCGCCTCGCGCCCCGCAGTCATT  -293
-292  GTCTTCGGGGAGAATTTTCTGGGGGACCTTCAGCGGATTGTCTGCGTGCAACAGGAGGTTGAGATTAAAGATGCAGCACTTGAAACAAATTATGAGGCAA  -193
                                                   -143      -132
-192  ATCAGCCAATGCAGGCCAGAGGCGGGGCCAGCAACACCCCGCAATTAGGCTGAACTCTCCGTGAACTGACTCATCTGAGGGAGGTGAGGGCTGCGCGCGC   -93
               -82                                                                            -1
-92   TCCTGCAACCTATAAAGGGTCCGCGGGGAGCGCGCGGGACCCCAGTCTGTGGGAGACGTTCACCTGAGCCGCAGCGCCCCGCACACCGCGACATG
```

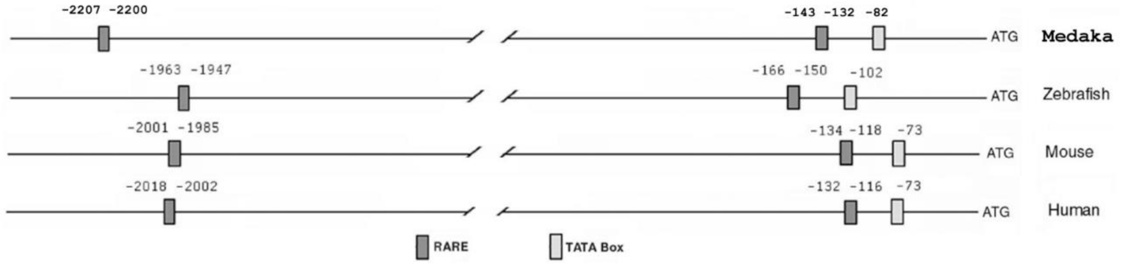

Supplementary Figure 4. Sequence of medaka promotor region of *cyp26a1*. RA responsive elements (RARE) in the *cyp26a1* medaka promoter region. Those elements are conserved in the promoter of this gene in different species. Promoters sequences were retrived from GenBank (http://www.ncbi.nlm.nih.gov). Scheme adapted from ref.[32].
